# Identifying a baicalein-related prognostic signature contributes to prognosis prediction and tumor microenvironment of pancreatic cancer

**DOI:** 10.3389/fimmu.2023.1223650

**Published:** 2023-07-28

**Authors:** Citing Zhang, Defeng Lei, Yan Zhou, Tongning Zhong, Xuefei Li, Weipeng Ai, Biao Zheng, Jikui Liu, Yicui Piao, Zilong Yan, Zhengquan Lai

**Affiliations:** ^1^ Department of Pharmacy, Shenzhen University General Hospital, Shenzhen University Clinical Medical Academy, Shenzhen University, Shenzhen, Guangdong, China; ^2^ Department of Hepatobiliary Surgery, Peking University Shenzhen Hospital, Shenzhen, Guangdong, China; ^3^ Department of Obstetrics & Carson International Cancer Research Center, Shenzhen University General Hospital and Shenzhen University Clinical Medical Academy, Shenzhen, Guangdong, China; ^4^ College of Stomatology, Dalian Medical University, Dalian, Liaoning, China; ^5^ Department of Surgery, The First Dongguan Affiliated Hospital, Guangdong Medical University. Dongguan, Guangdong, China; ^6^ Department of Critical Care Medicine, National Cancer Center, Cancer Hospital & Shenzhen Hospital, Chinese Academy of Medical Sciences and Peking Union Medical College, Shenzhen, Guangdong, China

**Keywords:** pancreatic cancer, baicalein, FGFBP1, cancer-associated fibroblast, liver metastasis, bioinformatics, high-throughput RNA dequencing, prognostic model

## Abstract

Pancreatic ductal adenocarcinoma (PDAC) is one of the most malignant and lethal human cancers in the world due to its high metastatic potential, and patients with PDAC have a poor prognosis, yet quite little is understood regarding the underlying biological mechanisms of its high metastatic capacity. Baicalein has a dramatic anti-tumor function in the treatment of different types of cancer. However, the therapeutic effects of baicalein on human PDAC and its mechanisms of action have not been extensively understood. In order to explore the biological characteristic, molecular mechanisms, and potential clinical value of baicalein in inhibiting the metastatic capacity of PDAC. We performed several *in vitro*, *in vivo*, and *in silico* studies. We first examined the potential regulation of baicalein in the metastatic capacity of PDAC cells. We showed that baicalein could dramatically suppress liver metastasis of PDAC cells with highly metastatic potential in mice model. The high-throughput sequencing analysis was employed to explore the biological roles of baicalein in PDAC cells. We found that baicalein might be involved in the infiltration of Cancer-Associated Fibroblasts (CAF) in PDAC. Moreover, a baicalein-related risk model and a lncRNA-related model were built by Cox analysis according to the data set of PDAC from TCGA database which suggested a clinical value of baicalein. Finally, we revealed a potential downstream target of baicalein in PDAC, we proposed that baicalein might contribute to the infiltration of CAF via FGFBP1. Thus, we uncovered a novel role for baicalein in regulation of PDAC liver metastasis that may contribute to its anti-cancer effect. We proposed that baicalein might suppress PDAC liver metastasis via regulation of FGFBP1-mediated CAF infiltration. Our results provide a new perspective on clinical utility of baicalein and open new avenues for the inhibition of liver-metastasis of PDAC.

## Introduction

PDAC is an exceptionally incurable disease, which is becoming the leading cause of patients with cancer. It was estimated that PDAC will become the second leading cause of cancer-related deaths by 2030, result of its abysmal survival rate (1, 2). Thus, there is an unquestionable need for more effective PDAC treatments. As of today, the molecular mechanisms underlying PDAC high malignancy remain unclear. Tumor microenvironment (TME) is the internal environment surrounding the tumor containing immune cells, stromal cells, and host factors. Increasing evidence indicates an important role of the TME in tumor malignancy and progression (3, 4). Meanwhile, TME has become a central research field to explore novel therapeutic targets (4–6). Over previous years, TME has been demonstrated to play critical roles in PDAC progression (7). However, the underlying mechanism of TME-mediated malignancy and development of PDAC remains elusive. The microenvironment of PDAC is composed of cancer cells, stromal cells and extracellular components, which interact closely with pancreatic cancer cells to create a PDAC-promoting microenvironment for its proliferation or metastasis. The CAF is the most prominent and active cell within the PDAC stromal and is proposed the play critical roles in PDAC progression. Relatively, CAF is poorly characterized cells that variably impact tumor progression and development. Studies proposed that CAF-derived chemokines, cytokines, growth factors, miRNAs, and exosomes can instruct PDAC to promote its malignancy (8–10). Given the importance of CAFs in PDAC, plenty of studies attempts to specifically target the CAF to develop therapeutic approaches for treatment of PDAC (11, 12). Baicalein is a flavone, originally isolated from the roots of Scutellaria baicalensis (Huangqin), which was widely used in traditional Chinese medicine, with an established pharmacological potential including anti-bacterial, antiviral, anti-inflammatory, and anti-oxidant activities (13–17). Meanwhile, in recent years, studies in anti-cancer field have shown anti-cancer effects of baicalein against various cancers (18–21). So far, the effect and mechanisms action of baicalein remain largely unknown. In particular, means of exploring the efficacy of baicalein against PDAC metastatic potential is limited. Thus, in this study, we aimed to understand the significance of the baicalein on PDAC metastatic potential and to address the possibility that baicalein might contribute to inhibition of PDAC metastatic ability via CAF-mediated PDAC progression.

In clinical decision-making, risk prediction models have become increasingly important in recent years (22). The risk prediction models are often used to evaluate the risk of patients with various diseases. It is proposed that the risk model could be used to study the correlation between future or unknown clinical outcomes and baseline health status of patients with diseases. In the study, we constructed two risk models for prediction of PDAC using baicalein-associated genes and FGFBP1-associated lncRNAs which might be a potential downstream target of baicalein.

## Materials and methods

### Cell culture and reagent

The human normal pancreatic ductal epithelial HPNE cell and human pancreatic cancer cell lines including PANC-1, SUIT-2, highly metastatic potential SUIT-2 cells (23) were maintained in DMEM medium (Gibco, Grand Island, NY, USA) supplemented with 10% fetal bovine serum (Gibco), and 1% Pen/Strep. All cells were grown at 37 °C in a humidified 5% CO_2_ atmosphere. For *in vitro* treatment, baicalein (HY-N0196, MCE) was dissolved in DMSO and diluted in DMEM, the final concentration of DMSO was less than 0.1%.

### *In vivo* experiments

The liver metastasis xenograft mouse model was described before (24), 1 x 10^6^ HM-SUIT-2 cells were resuspended in 100 µl of PBS and splenic transplanted into each of 10 nude mice (4 weeks old BALB/c athymic female), after 5 mins of hemostasis, spleens were then resected. 2 weeks after implantation, mice were randomized into two groups (n = 5 per group) for intraperitoneal treatment with PBS (control) or baicalein (25 mg/kg) every day for 1 week. At the end of drug administration, the liver lesions were obtained and evaluated.

### Cell viability assay

The cell viability was examined using CCK8 assay (C0038, Beyotime, China). The cells were seeded in a 96-well plate and cultured overnight. The cells were then treated with different concentrations of baicalein for 24 or 48h. Subsequently, cells were incubated with CCK8 reagent for 1h, followed by absorbance detection at 450 nm.

### High throughput sequencing

The total RNA was extracted from cells using RTIzol RNA isolation reagent according to the manufacturer’s instructions (AG, China). The transcriptome was evaluated by RNA sequencing (RNA-seq) which was performed at Novogene Bioinformatics Institute (China).

### Gene ontology analysis

The gene ontology analysis was performed and visualized using metascape webtool or R Clusterprofiler package.

### Modelling and evaluation of gene markers

The univariate COX regression analysis was carried out to screen the relevant genes associated with tumor prognosis using the RNA expression pattern and their clinical information obtained from TCGA database. The prognosis-related relevant genes were further subjected to least absolute shrinkage and selection operator (LASSO), and then constructed the prognostic risk model. Next, the patients with PDAC from TCGA database were divided into two groups according to the median risk score of the risk model. The ROC curves were generated using survival, survminer, and timeROC packages using R language.

### Quantitative RT−PCR

The total RNA was extracted from cells using RTIzol RNA isolation reagent according to the manufacturer’s instructions (AG, China). cDNA was synthesized using the 5 x EVO M-MLV RT Master Mix (AG, China). The Quantitative Real-time PCR (RT-PCR) was conducted using the SYBR Green Pro Taq HS premix (AG, China). The RT-PCR was performed on the platform of LightCycler480IIPCR system (Roche, Switzerland). The RT-PCR conditions were shown as follow: 95 °C 1 min, 95°C 10 s, 60°C 30s for 40 cycles. The primers were as follows: GAPDH forward primer:5′-AGGTGAAGGTCGGAGTCAACG-3′;GAPDH reverse primer: 5′-AGGGGTCATTGATGGCAACA-3′;FGFBP1 forward primer: 5′-ATGGACTTCACAGCAAAGTGGT-3′;FGFBP1 reverse primer: 5′-GGCTTGGTCTTTGGTGACAAAC′; Relative quantification of genes was analyzed inaccordance with the 2-ΔΔCt method (ΔΔCt = ΔCt [treated]-ΔCt [control]).

### Protein docking

To assess the binding affinities and interactions between the baicalein and FGFBP1 protein, we performed protein-ligand docking using AutodockVina 1.2.2, a silico protein-ligand docking software. For docking analysis, all protein and molecular files were converted into PDBQT format with all water molecules excluded and polar hydrogen atoms were added. The grid box was centered to cover the domain of each protein and to accommodate free molecular movement. The grid box was set to 30 Å × 30 Å × 30 Å, and grid point distance was 0.05nm. Molecular docking studies were performed by Autodock Vina 1.2.2 (25, 26).

## Results

### Baicalein might contribute to metastatic potential of PDAC *via* regulating of TME

**Figure 1:**
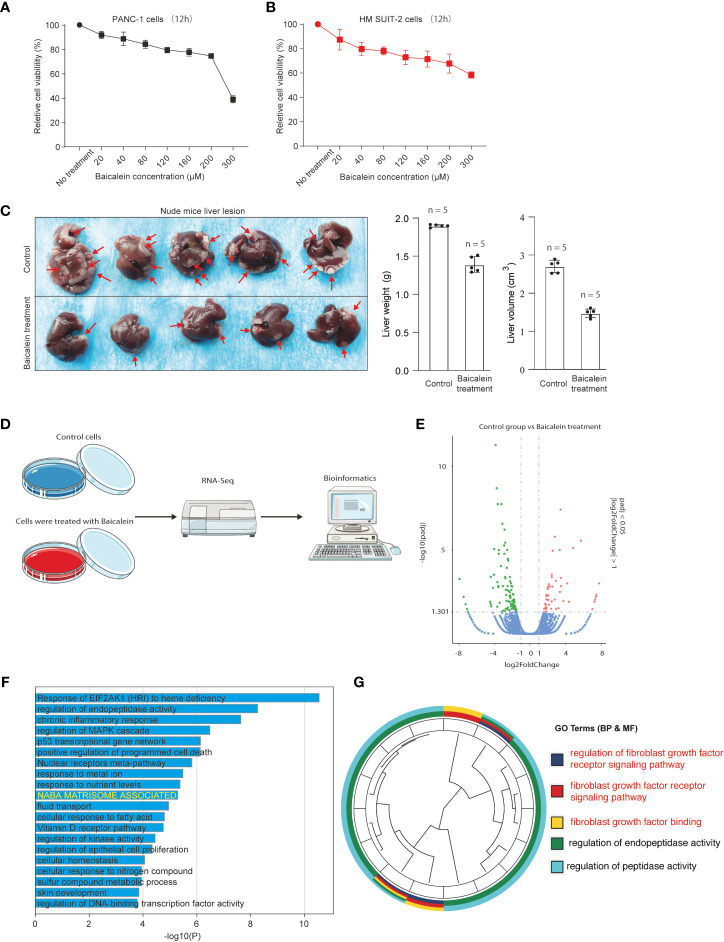
**(A)** Cell viability assay of PANC-1 cells treated with different concentrations of baicalein for 12h. **(B)** Cell viability assay of HM-SUIT2 cells treated with different concentrations of baicalein for 12h. **(C)** The liver lesion of PDAC cells in nude mice without baicalein or following treatment with baicalein. **(D)** The RNA-Seq analysis using HM SUIT-2 cells treated without baicalein or following treatment with baicalein. **(E)** Differentially expressed gene screening between control group and baicalein treated group. **(F)** GO analysis using metascape web tool. **(G)** GO analysis using cluster profile package in R language.

To explore the biological effects of baicalein in PDAC, we first examined the effect of baicalein on cell viability in different PDAC cells. PANC-1 cells were derived from pancreatic carcinoma of ductal cell origin with poor differentiation abilities (27, 28). Highly Metastatic potential SUIT-2 (HM-SUIT-2) cells were isolated from wild-type SUIT-2 cells through *in vivo* selection from mice liver metastasis (23). We treated PANC-1 cells and HM-SUIT-2 cells with different concentrations indicated for 12 h. Then, the cell viability was assessed as shown in **Figures 1A, B**. We observed that baicalein could suppress cell viability of PANC-1 cells (**Figure 1A**). Whereas suppression of cell viability in HM-SUIT-2 cells was relatively weak compared with PANC-1 cells. We did not examine a proper half-maximal inhibitory concentration of baicalein in HM-SUIT-2 until 300 µM (**Figure 1B**). To further assess the effect of baicalein on liver metastasis formation, we established the splenic xenograft mice model by implantation of HM-SUIT-2 cells. 2 weeks after cell transplantation, baicalein (25 mg/kg) was administered via intraperitoneal injection once a day. After 1-week treatment, the liver metastasis lesions were assessed using mice liver. As shown in **Figure 1C**, the red arrows indicated the liver metastases. We observed reductions of liver weight and volume after baicalein treatment (**Figure 1C** and **Figure S1**). The result revealed that the liver metastases were decreased in baicalein treatment group compared with control group. Combining the *in vitro* and *in vivo*, baicalein could suppress the liver metastasis of HM-SUIT-2 cells in mice, while the effect of baicalein on viability of HM-SUIT-2 was not significant. We therefore curious about the mechanism underlying baicalein on the inhibition of liver metastatic potential. To further explore the biological effect of baicalein on HM-SUIT-2 cells, we then conducted the high-throughput sequencing of RNA (RNA-Seq) to compare the RNA expression pattern between no treated HM-SUIT-2 cells and baicalein treated HM-SUIT-2 cells. The bioinformatic analysis was performed to screen the differentially expressed genes according to |logFC| > 1 and p < 0.05 (**Figures 1D, E**). Subsequently, Gene Ontology (GO) analysis was performed using the baicalein-associated differentially expressed genes. As shown in **Figure 1F**, the GO results from metascape web tool (29) indicated that the baicalein-associated differentially expressed genes were highly enriched in extracellular matrix relevant pathway (**Figure 1F**, yellow). To further validate the result, we again analyzed the differentially expressed genes using cluster profile package in R language (30, 31). The GO analysis indicated that the baicalein-associated differentially expressed genes were involved in fibroblast growth factor binding pathway (**Figure 1G**). The results above enable us to put forward a hypothesis that baicalein might contribute to TME via CAF regulation.

### The prognostic values of baicalein-associated genes in PDAC

**Figure 2:**
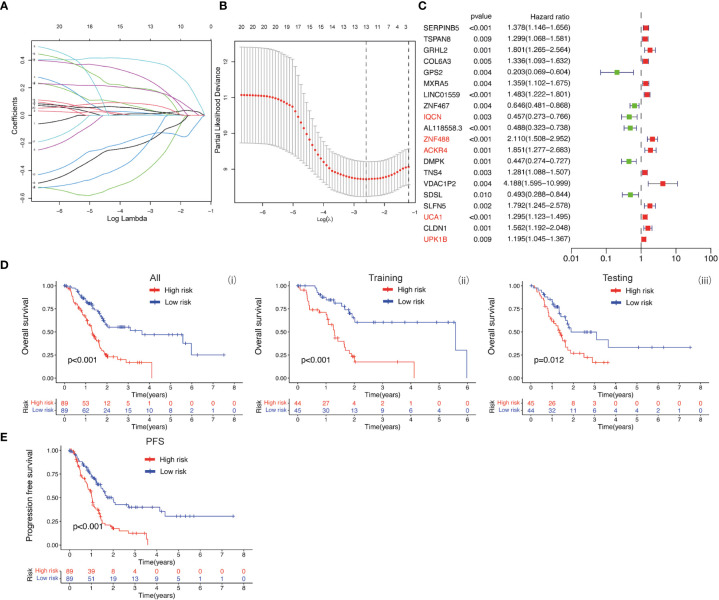
**(A, B)** LASSO and COX regression analysis. **(C)** COX univariate analysis. **(D)** (i) Kaplan-Meier curve of overall survival between patients in high-risk group and low-risk group. (ii) Kaplan-Meier curve of survival between patients in high-risk group and low-risk group in Training cohort. (iii) Kaplan-Meier curve of clinical outcomes between patients in high-risk group and low-risk group in Testing cohort. **(E)** Kaplan-Meier curve of progression free survival between patients in high-risk group and low-risk group.

**Figure 3:**
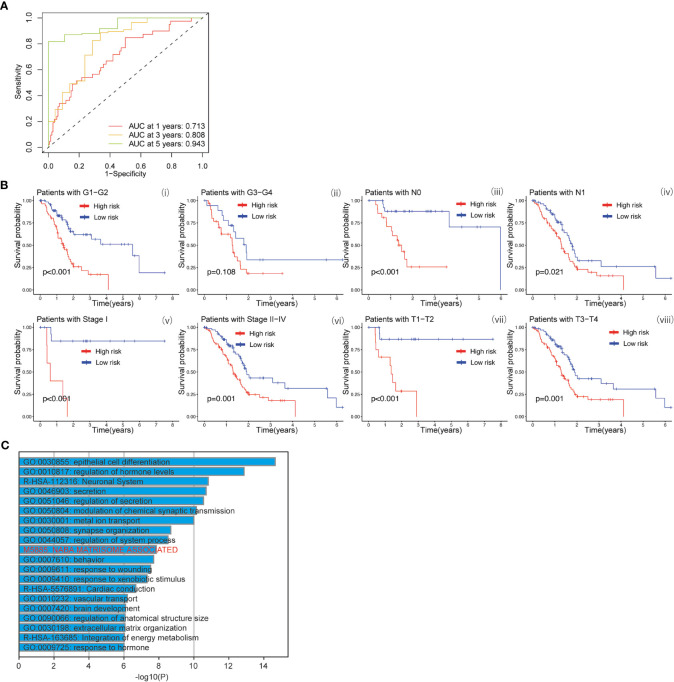
**(A)** ROC curves of the prognostic model for predicting the 1-year, 3-years, and 5-years survival. **(B)** Kaplan-Meier curve of clinical outcomes between patients in high-risk group and low-risk group according to different clinical features. **(C)** GO analysis using metascape web tool.

The baicalein-associated differentially expressed genes were obtained according to |logFC| > 1 and p < 0.05 using RNA-Seq in HM-SUIT-2 cells. Meanwhile, the PDAC gene expression profiles and their clinical information were obtained from TCGA. To further investigate the prognostic values of baicalein-associated genes in PDAC, we performed the LASSO and Cox regressions using baicalein-associated genes and their expression profiles, and clinical information from TCGA database. Depending on the results of the analysis and screening above, we constructed a baicalein-associated prognostic risk model for PDAC using the baicalein-associated genes (IQCN, ZNF488, ACKR4, UCA1, and UPK1B) (**Figures 2A–C**). We divided the patients with PDAC into high-risk group and low-risk group according to the median risk score. Then the survival analysis was performed between the high- and low-risk groups. As shown in **Figure 2D** (i), the overall survival in high-risk group was significantly low than that in the low-risk group. The patients with PDAC were divided into the training group and testing group, then the survival conditions were examined in training and testing groups according to the risk score. As shown in **Figure 2D** ii and iii, Kaplan-Meier curves showed that patients with PDAC in high-risk groups were accompanied by a relatively lower survival time compared with patients in low-risk groups in training and testing cohort. The same result was observed in progression-free survival (**Figure 2E**). The results above indicated that the risk model for PDAC using the baicalein-associated genes was associated with the survival condition of PDAC. To further examine the clinical values of the established baicalein-associated prognostic risk model, we draw ROC curves to evaluate the diagnostic values of the baicalein-associated prognostic risk model. As shown in **Figure 3A**, we observed an increasing AUC value in ROC analysis using the risk score. The ROC of the risk score showed AUC values of 0.731, 0.808, and 0.943 at 1, 3, and 5 years respectively (**Figure 3A**). We further examined the survival conditions of patients with PDAC between the high- and low-risk groups with different clinical features. As shown in **Figure 3B**, we observed that the survival condition of patients with PDAC in low-risk group was better than in high-risk group in both histological grade 1&2 and grade 3&4 (**Figure 3B** i and ii). The survival time of patients with PDAC in low-risk group was longer than the patients in high-risk group in N0 and N1 (**Figure 3B** iii and iv). We also examined the patient survival condition in both early stage (stage I, T1&T2) (**Figure 3B** v and vii) and late stage (stage II-IV, T3&T4) (**Figure 3B** vi and viii). It is consistent with the observation above. The patients in low-risk group were accompanied by better survival compared with high-risk group in both early stage and late stage of PDAC (**Figure 3B** v, vi, vii, and viii). The results above revealed that the baicalein-associated risk model could indicate patient survival across different severities of PDAC. To further explore the biological significance of the baicalein-associated risk model. We carried out GO analysis to detect the biological meaning involved in the low- and high-risk groups using the differentially expressed genes between the two groups. The GO analysis was performed using metascape web tool. As shown in **Figure 3C**, the differentially expressed genes from baicalein-associated risk model were highly enriched in the NABA MATRISOME ASSOCIATED pathway which belongs to extracellular matrix-associated pathway that is involved in TME. The results revealed that the baicalein-associated risk model is involved in TME.

### The potential clinical value of FGFBP1 in PDAC

**Figure 4:**
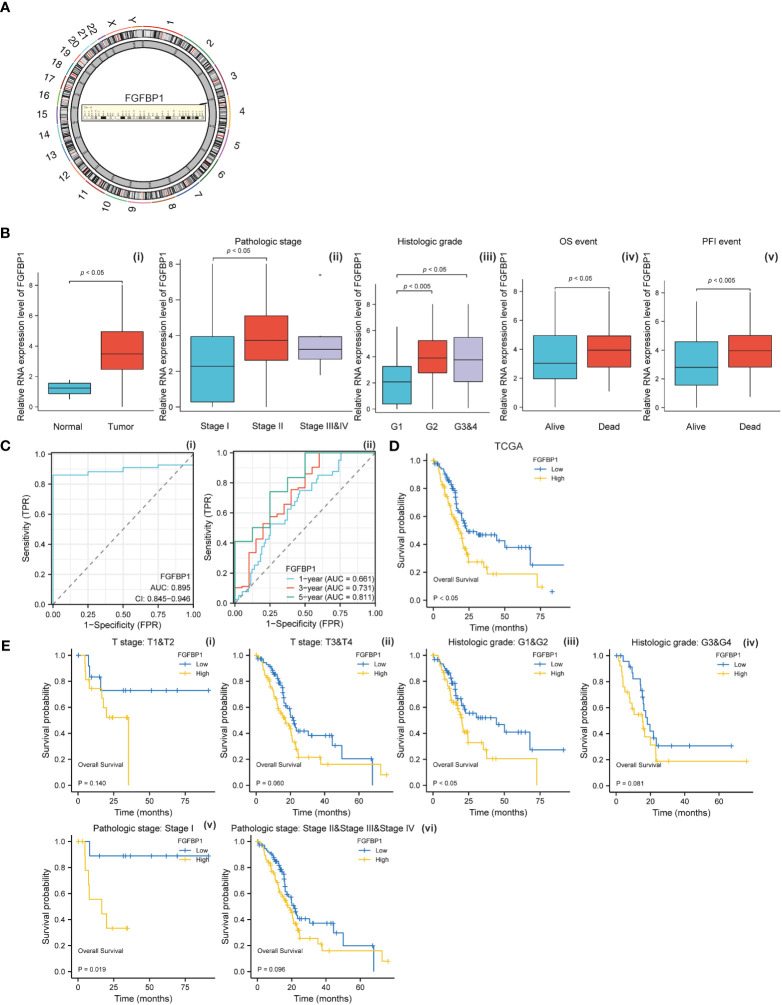
**(A)** Chromosomal location of FGFBP1. **(B)** (i) Relative expression level of FGFBP1 between normal and PDAC tissues. (ii) Relative expression level of FGFBP1 in stage I, stage II, and stage III&IV. (iii) Relative expression level of FGFBP1 in histological grade 1, histological grade 2, and histological grade 3&4. (iv) Relative expression level of FGFBP1 for overall survival between alive and dead patients with PDAC (v) Relative expression level of FGFBP1 for progression free survival between alive and dead patients with PDAC. **(C)** (i) ROC curve of FGFBP1 between normal and PDAC tissues. (ii) ROC curves of FGFBP1 for predicting the 1-year, 3-years, and 5-years survival. **(D)** Kaplan-Meier curve of clinical outcomes between patients in high- and low-FGFBP1 expression group using dataset from TCGA database. **(E)** Kaplan-Meier curve of clinical outcomes between patients in high- and low-FGFBP1 expression groups according to different clinical features.

To investigate the biological effects of baicalein in PDAC, we performed the RNA-Seq to compare the RNA expression pattern between no treated HM-SUIT-2 cells and baicalein treated HM-SUIT-2 cells. CAFs compose a significant component of PDAC TME. Based on the correlation between baicalein and PDAC TME, we next screened the baicalein-targeting genes that are associated with CAF infiltration. According to the differentially expressed genes that resulted from baicalein treatment. We observed that the FGFBP1 was significantly downregulated after baicalein treatment in HM-SUIT-2 cells. FGFBP1 is a gene that encodes the secreted fibroblast growth factor carrier protein which is an extracellular chaperone molecule for fibroblast growth factors, and has been proposed to enhance fibroblast growth factor-mediated biochemical events (**Figure 4A**) (32). Compared to its low expression in normal tissues, FGFBP1 expression level is higher in various cancers (33). To examine the clinical values of FGFBP1, we examined the correlation between FGFBP1 expression and clinical features using datasets from TCGA. As shown in **Figure 4B**, the expression level of FGFBP1 was higher in tumor tissues compared with normal tissues (**Figure 4B** i). Meanwhile, we observed that the expression level of FGFBP1 was increasing accompanied by the pathological stage and histological grade (**Figure 4B** ii and iii). As shown in **Figure 4B** (iv-v), in the overall survival (OS) events and progression-free interval (PFI) events, we observed that FGFBP1 expression was higher in dead than in alive patients, suggesting that low expression of FGFBP1 seemed to have a better prognosis. To further evaluate the clinical value of the FGFBP1, we draw ROC curves to evaluate the diagnostic values of FGFBP1 in PDAC. As shown in **Figure 4C**, the AUC value for FGFBP1 to discriminate between tumor and normal was 0.895. Meanwhile, we observed an increasing AUC value in ROC analysis using the risk score. The ROC of the risk score showed AUC values of 0.661, 0.731, and 0.811 at 1, 3, and 5 years respectively (**Figure 4C**, ii). We further examined the survival conditions of patients with PDAC between the high- and low-expression groups of FGFBP1. As shown in **Figure 4D**, we observed that the survival condition of patients with PDAC in low-expression group of FGFBP1 was better than in high-expression group of FGFBP1 in TCGA database. We also examined the survival condition between high- and low-expression groups of FGFBP1 with different clinical features. As shown in **Figure 4E**, we observed that the survival condition of patients with PDAC in low-expression group of FGFBP1 was better than in high-expression group of FGFBP1 in both T stage 1&2 and T stage 3&4 (**Figure 4E** i and ii). Same results were observed in both histological grade 1&2 and grade 3&4 (**Figure 4E** iii and iv). The survival time of patients with PDAC in low-expression group of FGFBP1 was longer than the patients in high-expression group of FGFBP1 in both pathogogic stage I and pathogogic stage II, III and IV (**Figure 4E** v and vi). The results above revealed the clinical values of FGFBP1 in PDAC across different clinical features.

### Treatment of bicalein causes the downregulation of FGFBP1 expression in PDAC *in vitro*

**Figure 5:**
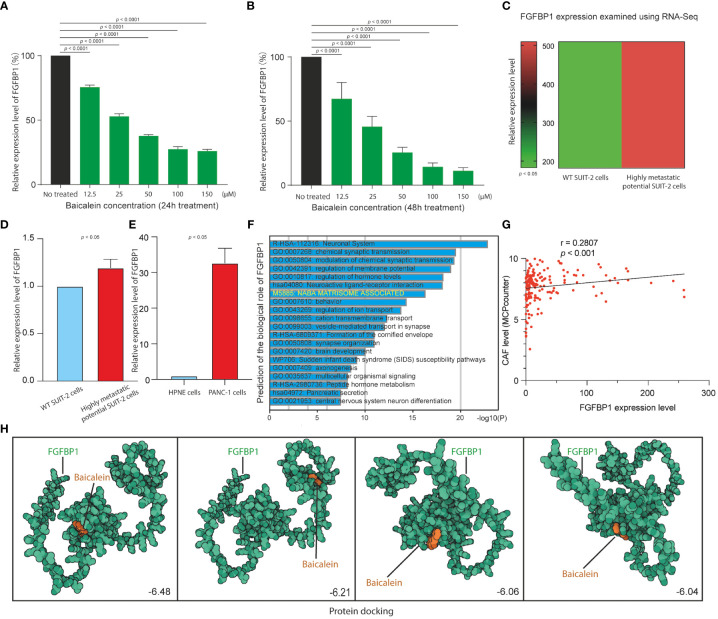
**(A)** FGFBP1 expression level in HM-SUIT cells after baicalein treatment for 24h determined by RT-PCR. **(B)** FGFBP1 expression level in HM-SUIT cells after baicalein treatment for 48h determined by RT-PCR. **(C)** FGFBP1 expression level in wild tye SUIT-2 cell and HM-SUIT cells determined by microarray. **(D)** FGFBP1 expression level in wild tye SUIT-2 cell and HM-SUIT-2 cells determined by RT-PCR. **(E)** FGFBP1 expression level in HPNE cell and PANC-1 cells determined by RT-PCR. **(F)** GO analysis using differentially expressed genes between FGFBP1 high- and low-expression samples obtained from TCGA database. **(G)** Correlation analysis between CAF infiltration level and FGFBP1 expression level using dataset from TCGA database. The infiltration level of CAF was evaluated via MCP-counter package using R language. **(H)** Protein-ligand docking to predict the binding affinities and interactions between the baicalein and FGFBP1 protein.

We next examined the correlation between baicalein and FGFBP1 expression in PDAC cells to determine the effects of baicalein on the FGFBP1 expression pattern. We performed quantitative real-time PCR (qRT-PCR) to examine FGFBP1 RNA expression level after baicalein treatment in PDAC cells. As shown in **Figures 5A, B**, we observed dramatic downregulations of baicalein in a dose-dependent manner for 24 and 48h treatment, suggesting that baicalein could downregulate FGFBP1 expression level in a dose-dependent manner in PDAC cells. Meanwhile, to explore the role of FGFBP1 in PADC metastatic potential, we also examine the expressions of FGFBP1 in wild-type (WT) SUIT-2 and highly metastatic potential SUIT-2 cells using RNA-Seq data (23). The results indicated that FGFBP1 expression level upregulated in highly metastatic potential SUIT-2 cells (**Figure 5C**). To validate the upregulation of FGFBP1 in highly metastatic potential SUIT-2 cells, we examined FGFBP1 expression using RT-PCR in WT SUIT-2 and highly metastatic potential SUIT-2 cells (**Figure 5D**). The expression levels of FGFBP1 in normal pancreatic epithelial cells (HPNE cells) and pancreatic tumor cells (PANC-1 cells) were examined, we found that FGFBP1 expression was higher in PANC-1 cells than in HPNE cells (**Figure 5E**). The results above reveal that the upregulation of FGFBP1 is accompanied by the high metastatic capability of PDAC cells. To further predicted biological function of FGFBP1, we divided the PDAC samples into FGFBP1-high and low expression groups according to the expression dataset from TCGA. We then performed GO analysis using different expression genes between FGFBP1-high and low-expression groups. As shown in **Figure 5F**, the different expression genes between FGFBP1-high and low-expression groups were associated with the NABA MATRISOME ASSOCIATED pathway with is a TME-related pathway. Meanwhile, we next performed quantification of CAFs in PDAC using the dataset obtained from TCGA database. The MCPcounter package was used for the analysis. Then the correlation coefficient between FGFBP1 and CAFs immune infiltration was calculated. As shown in **Figure 5G**, we observed that FGFBP1 was correlated with infiltration of CAFs (r = 0.2807, *p* < 0.001). To further determine the affinity or interaction between baicalein and FGFBP1 at protein level. The docking between baicalein and FGFBP1 protein was performed using the docking platform. The binding poses and interactions between baicalein and FGFBP1 protein were obtained with Autodock Vina v.1.2.2 and the binding energy for each interaction was generated. The docking results revealed the binding site of baicalein interacting with FGFBP1 protein (**Figure 5H**). The results above revealed that the regulation of baicalein on FGFBP1 is not only at RNA transcription or stability level but also likely at the protein interaction level. Studies proposed that FGFBP1-mediated FGF signaling pathway contributes the stroma on PDAC (34). Given the observed correlation between baicalein and TME of PDAC, and the baicalein-mediated downregulation of FGFBP1, as well as the role of FGFBP1 in CAF infiltration. we present the hypothesis that baicalein might contribute to TME of PDAC via FGFBP1 mediated CAF infiltration.

### The prognostic values of FGFBP1-associated lncRNA in PDAC

**Figure 6:**
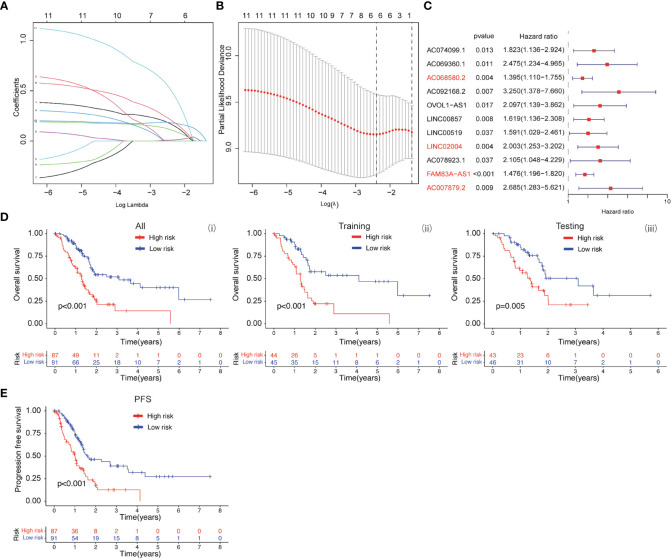
**(A, B)** LASSO and COX regression analysis. **(C)** COX univariate analysis. **(D)** (i) Kaplan-Meier curve of overall survival between patients in high-risk group and low-risk group. (ii) Kaplan-Meier curve of survival between patients in high-risk group and low-risk group in Training cohort. (iii) Kaplan-Meier curve of clinical outcomes between patients in high-risk group and low-risk group in Testing cohort. **(E)** Kaplan-Meier curve of progression free survival between patients in high-risk group and low-risk group.

**Figure 7:**
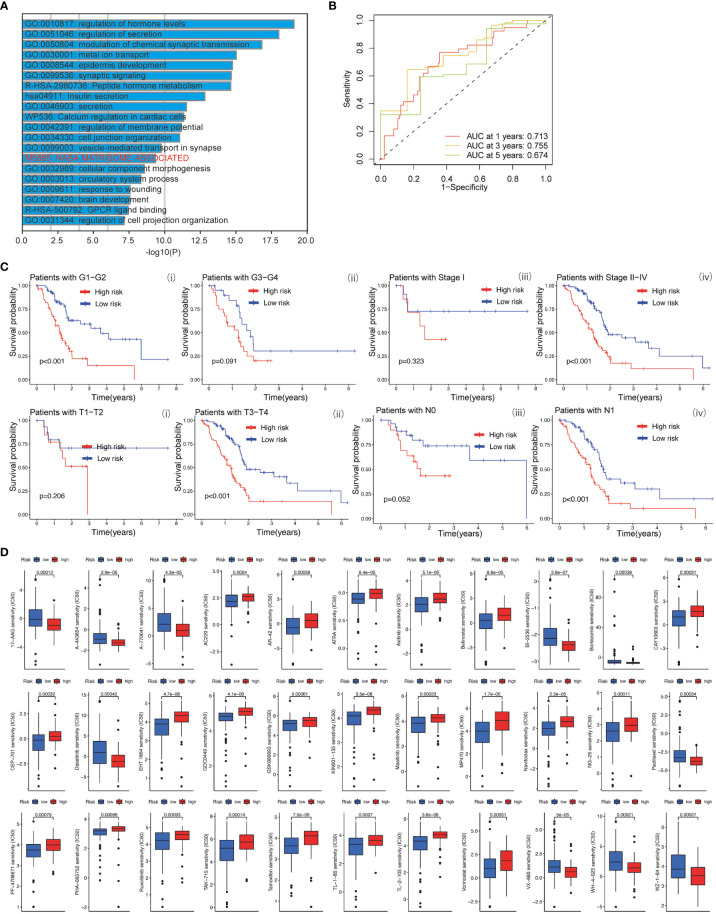
**(A)** GO analysis using metascape web tool. **(B)** ROC curves of the prognostic model for predicting the 1-year, 3-years, and 5-years survival. **(C)** Kaplan-Meier curve of clinical outcomes between patients in high-risk group and low-risk group according to different clinical features. **(D)** Screening of chemotherapeutic agents in high-risk group and low-risk group: The sensitivity (IC50) of screened chemotherapeutic agents between high-risk group and low-risk group.

Based on the clinical value of FGFBP1 in PDAC, we next investigated the clinical value of FGFBP1-associated lncRNA in PDAC. We extracted the lncRNAs expression matrix of patients with PDAC from TCGA database. We then identified the FGFBP1 lncRNAs according to criteria of correlation > 0.2 and *p* < 0.05. Next, we performed the LASSO and univariate Cox regressions using FGFBP1-associated lncRNAs and their expression profiles, and clinical information from TCGA database. The prognosis-related lncRNAs were further subjected to construct an FGFBP1-associated lncRNA prognostic risk model for PDAC using the FGFBP1-associated lncRNAs (AC068580.2, LINC02004, FAM83A-AS1, and AC007879.2) (**Figures 6A–C**). To validate the prediction reliability of the FGFBP1-associated lncRNA prognostic risk model for PDAC, the patients with PDAC were divided into high-risk group and low-risk group according to the median risk score. We then carried out the survival analysis between high-risk group and low-risk group. Kaplan-Meier curves showed that the overall survival time of the low-risk group was longer than the high-risk group (**Figure 6D** i). Similar results were also observed in the training and testing groups, the patients in the low-risk group accompanied by a better survival condition compared with the patients in high-risk group (**Figure 6D** ii and iii). Consistent with the results above, the patients in the low-risk group showed a better survival condition than the patients in high-risk group for progression-free survival (**Figure 6E**). We next assessed the biological significance associated with FGFBP1-associated lncRNA prognostic risk model. We performed GO analysis to detect the biological meaning involved in FGFBP1-associated lncRNA prognostic risk model using the differentially expressed genes between the low- and high-risk groups. As shown in **Figure 7A**, we found that the differentially expressed genes were enriched in the NABA MATRISOME ASSOCIATED pathway which belongs to extracellular matrix-associated pathway that is involved in TME. The results above suggest that FGFBP1-associated lncRNA prognostic risk model is associated with the TME of PDAC. To further evaluate the prognostic value of the FGFBP1-associated lncRNA prognostic risk model, we performed ROC analysis using the risk score. The AUC values at 1, 3, and 5 years in the time-dependent ROC cure were 0.713, 0.755, and 0.674, respectively (**Figure 7B**). We next did a subgroup survival analysis based on different clinical features. The Kaplan-Meier survival analyses for PDAC patients with different histological grades, clinical stages, T stages, and N stages were performed (**Figure 7C**). The results revealed that patients with PDAC in high-risk group were accompanied by a trend of worse conditions in both early and advanced stages of PDAC. To explore the clinical utility of the FGFBP1-associated lncRNA prognostic risk model for PDAC, we further screened the potential therapeutic drugs that showed different sensitivities between high- and low-risk groups (*p < 0.05*). As shown in **Figure 7D**, the results revealed that 33 potential chemotherapeutic agents were more responsive to the risk score of FGFBP1-associated lncRNA prognostic risk model between high- and low- risk groups.

## Discussion

PDAC is a common and malignant disease with a poor prognosis that is currently difficult to be detected. Therefore, it is crucial to explore new therapeutic treatments and targets for PDAC. The late diagnosis and metastasis are mainly responsible for the high mortality of PDAC patients. Patients with PDAC are always accompanied by the potency to metastasize early. Due to the strong metastatic potential of PDAC, strategies to suppress its metastatic-related functions will have significant clinical implications for PDAC treatment. Studies proposed that the TME of PDAC is critical for its progression. The TME in PDAC contributes to tumor growth, invasion, and metastasis in a multifaceted way. Therefore, TME is an emerging source of novel therapeutic targets in PDAC treatment (35). The PDAC TME contains abundant stromal cells including CAF, tumor-associated macrophage, and endothelial cells, that often lead to desmoplastic reaction (36). CAFs are the major contributors to the stromal evolution in PDAC which are the most dynamic stromal cells and characterized as accomplices and promoters for PDAC malignancy.

In recent years, exploration of therapeutic targets for tumors is a hot topic for cancer research. Baicalein, a natural compound widely used in Chinese herbal medicine, has been reported to be involved in the regulation of biological functions in various cancer (18–20). However, its effect on PDAC chemotherapy is still largely unknown. The aim of this study is to assess the anti-metastatic potential of PDAC. It was found that baicalein could decrease the liver metastatic ability of PDAC cells in the transplantation mice model. The liver colony formation ability of PDAC cells in mice model was decreased after baicalein treatment. Furthermore, the RNA-Seq analysis and bioinformatic analysis revealed that baicalein was involved in extracellular-related regulation and might contribute to stromal cell infiltration of PDAC. In addition, we proposed an FGFBP1 gene that might be the downstream target of baicalein. The results revealed that baicalein could dramatically downregulate FGFBP1 expression in a dose-depend manner. Meanwhile, protein-docking analysis showed that baicalein could interact with FGFBP1 at protein level. The prognostic value of FGFBP1 in PDAC was validated using dataset obtained from TCGA database and ICGA database. Finally, we constructed two prognostic prediction models for patients with PDAC using the baicalein-associated genes and FGFBP1-associated lncRNA.

The fibroblast growth factors (FGF) are essential for tumor progression and involved in a wide range of biological functions including cell proliferation, in various tumors (37, 38). Studies proposed that CAFs can be activated by FGF and cytokines (38). The FGFBP1 was characterized as a secreted fibroblast growth factor-binding protein as a chaperone molecule. It was proposed that FGFBP1 could enhance the functions of fibroblast growth factor by releasing FGFs from the extracellular matrix (39). Downregulation of FGF expression was detected in CAFs cocultured with PDAC cells with FGFBP1 abrogation suggesting the role of FGFBP1 in FGF-mediated CAF regulation (40). In the study, we employed high-throughput RNA sequencing for novel target screening of baicalein. We first link the baicalein with FGFBP1-mediated TME regulation in PDAC. Here, we provide evidence that FGFBP1 is a downstream target of baicalein. Meanwhile, we also demonstrated the clinical value of baicalein in PDAC therapy via FGFBP1 mediated TME regulation which provides new ideas and strategies for the clinical treatment of PDAC. The prognostic models proposed in the study offer therapeutic value for PDAC prognosis.

## Data availability statement

The datasets presented in this study can be found in online repositories. The names of the repository/repositories and accession number(s) can be found below: PRJNA967843 (SRA).

## Ethics statement

The animal study was reviewed and approved by The research was approved by the Experimental Animal Center in Guangzhou University of Chinese Medicine (Guangzhou, China).

## Author contributions

CZ, DL and YZ designed and performed research studies and wrote the manuscript. TZ and XL completed the experiments and data analysis. BZ, JL and WA analyzed and interpreted the data. YP, ZY and ZL supervised the whole study and critically revised the manuscript. All authors read and approved the final manuscript.

## Supplementary material

Supplementary Figure 1 The liver lesion of PDAC cells in nude mice without baicalein or following treatment with baicalein.
