# Supplementary figures and images for: Identifying a baicalein-related prognostic signature contributes to prognosis prediction and tumor microenvironment of pancreatic cancer
Source: Front Immunol. 2023 Jul 28;14:1223650. doi: 10.3389/fimmu.2023.1223650 (PMC10416623; doi:10.3389/fimmu.2023.1223650)

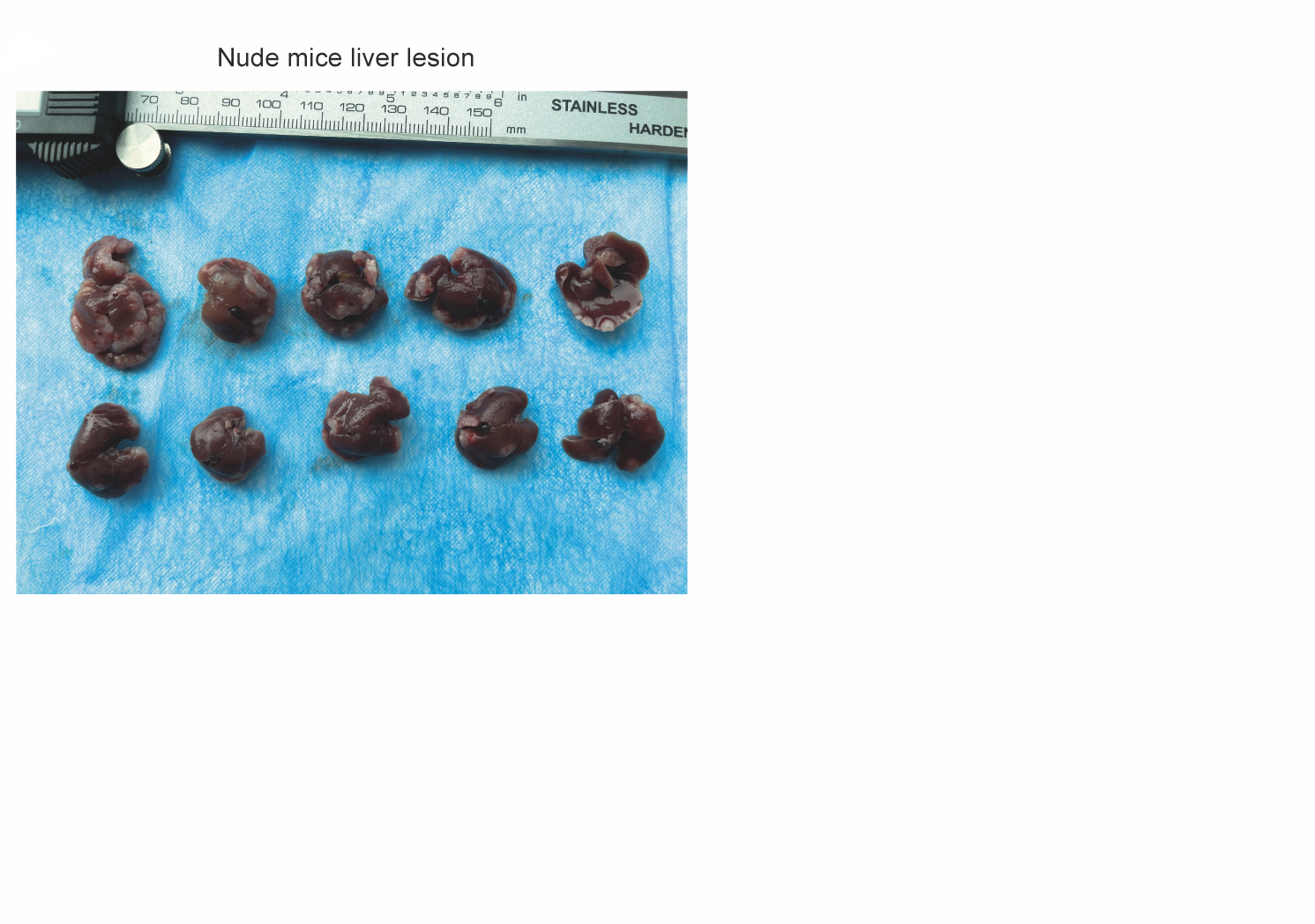

Supplement: Supplementary Figure 1 — The liver lesion of PDAC cells in nude mice without baicalein or following treatment with baicalein. [file Image_1.tif]
